# Transcriptomic Analysis Shows Decreased Cortical Expression of NR4A1, NR4A2 and RXRB in Schizophrenia and Provides Evidence for Nuclear Receptor Dysregulation

**DOI:** 10.1371/journal.pone.0166944

**Published:** 2016-12-16

**Authors:** Susan M. Corley, Shan-Yuan Tsai, Marc R. Wilkins, Cynthia Shannon Weickert

**Affiliations:** 1 Systems Biology Initiative, School of Biotechnology and Biomolecular Sciences, University of New South Wales, Sydney, New South Wales, Australia; 2 Schizophrenia Research Institute, Randwick, NSW, Australia; 3 Neuroscience Research Australia, Randwick, NSW, Australia; 4 School of Psychiatry, University of New South Wales Sydney, NSW, Australia; Chiba Daigaku, JAPAN

## Abstract

Many genes are differentially expressed in the cortex of people with schizophrenia, implicating factors that control transcription more generally. Hormone nuclear receptors dimerize to coordinate context-dependent changes in gene expression. We hypothesized that members of two families of nuclear receptors (NR4As), and retinoid receptors (RARs and RXRs), are altered in the dorsal lateral prefrontal cortex (DLPFC) of people with schizophrenia. We used next generation sequencing and then qPCR analysis to test for changes in mRNA levels for transcripts encoding nuclear receptors: orphan nuclear receptors (3 in the NR4A, 3 in the RAR, 3 in the RXR families and KLF4) in total RNA extracted from the DLPFC from people with schizophrenia compared to controls (n = 74). We also correlated mRNA levels with demographic factors and with estimates of antipsychotic drug exposure (schizophrenia group only). We tested for correlations between levels of transcription factor family members and levels of genes putatively regulated by these transcription factors. We found significantly down regulated expression of NR4A1 (Nurr 77) and KLF4 mRNAs in people with schizophrenia compared to controls, by both NGS and qPCR (p = or <0.01). We also detected decreases in NR4A2 (Nurr1) and RXRB mRNAs by using qPCR in the larger cohort (p<0.05 and p<0.01, respectively). We detected decreased expression of RARG and NR4A2 mRNAs in females with schizophrenia (p<0.05). The mRNA levels of NR4A1, NR4A2 and NR4A3 were all negative correlated with lifetime estimates of antipsychotic exposure. These novel findings, which may be influenced by antipsychotic drug exposure, implicate the orphan and retinoid nuclear receptors in the cortical pathology found in schizophrenia. Genes down stream of these receptors can be dysregulated as well, but the direction of change is not immediately predictable based on the putative transcription factor changes.

## Introduction

Schizophrenia is a serious psychiatric disorder adversely affecting the quality of life of a significant number of people [1]. Schizophrenia arises from a complex and varied set of environmental and genetic factors, which has made it very difficult to come to a clear understanding of the etiology of the condition, despite intensive scientific work in the area. However, it seems that a disease arising from the interplay of genes and environment is likely to involve the super family of nuclear receptors which are known to control gene expression depending on context.

A group of 48 transcription factors play a key role in transducing extracellular (environmental, metabolic, endocrine) signals into intercellular signals, resulting in changes in expression of target genes. The nuclear receptors (NRs) are grouped into 6 functionally related sub-families (NR1—NR6) and include the estrogen and androgen receptors (NR3A1/ESR1 and NR3C4/AR), the glucocorticoid and mineralocorticoid receptors (NR3C1/GR and NR3C2/MR), the retinoid receptors (NR1B/RARs and NR2B/RXRs), the vitamin D receptor (NR1I1/VDR), the peroxisome proliferator-activated (fatty acid) receptors (NR1C/PPARs) and the orphan nuclear receptors (NR4A sub-family)[2]. A number of these genes/transcripts have been implicated in schizophrenia, including the estrogen [3, 4] and the glucocorticoid receptors [5–8], the retinoid (vitamin A) receptors [9] and the NR4A (orphan) receptors [10]. The nuclear receptors generally dimerize to form either homodimers or heterodimers with other nuclear receptors and may be activated by multiple ligands. They are therefore part of a complex network of molecules essential for development and adaptive responses in the adult.

To gain a more complete picture of alterations in nuclear receptor alterations, we have focused this study on the NR4A sub-family of nuclear receptors (NR4A1 (Nurr 77 or NGF1B), NR4A2 (Nurr1), NR4A3 (Nor1)), and their dimerization partners, the retinoid X receptors (RXRA, RXRB, RXRG) and the retinoic acid receptors (RARA, RARB, RARG).

The RAR proteins are activated by all-trans retinoic acid while the RXR proteins are activated by by 9-cis retinoic acid, and other ligands such as the omega 3 unsaturated fatty acids and various synthetic compounds [11, 12]. NR4A1 and NR4A2, but not NR4A3 [13, 14], form active heterodimers with RXRA and RXRG [15, 16] and in this form can bind to the retinoid acid response elements in genomic DNA [15]. Whilst NR4A3 does not heterodimerize with the RXRs, it can interfere with the signaling from either the NR4A1-RXR or NR4A2-RXR complexes [14]. RXR dimerizes with several nuclear receptors including the retinoid receptors (RARs sub-family), the vitamin D receptor (VDR), the thyroid hormone receptors (T3Rs) and the lipid activated nuclear receptors (PPARs).

While NR4A2 has an important role in the cell body of dopaminergic neurons, the action of NR4A1 is more pronounced at target areas of the dopaminergic neurons, such as the prefrontal cortex. The NR4A1- RXR complex is suggested to function as an adaptive homeostatic regulator of dopamine neurotransmission [17]. Blockers of dopamine transmission, antipsychotics, can impact the expression of NR4A genes [18, 19] and gene ablation studies have demonstrated changes in response to antipsychotic medication in NR4A1 null mice [20, 21]. Thus, it is important to consider if the levels of antipsychotic drug levels correlate with levels of these nuclear receptor mRNAs in the brains of people with schizophrenia.

In this study, we have quantified and compared the mRNA expression of genes encoding nuclear receptors with a focus on those in the NR4A and RXR/RAR families. This study aims to determine if altered levels of the mRNA expressions in orphan nuclear receptors and retinoid receptors exist in the brains of people with schizophrenia using next generation sequencing and by real time quantitative polymerase chain reaction (RT-qPCR) in the DLPFC.

## Methods

### Post-mortem brain samples

Dorsal lateral prefrontal cortex (DLPFC) from thirty-seven schizophrenia/schizoaffective cases and thirty-seven controls was obtained from the New South Wales Tissue Resource Centre. Of the thirty-seven schizophrenia/schizoaffective cases, eight were on first generation antipsychotics only, twenty-two had predominantly received first generation antipsychotics, one was one second generation antipsychotics only, five had predominantly received second generation antipsychotics, and one received equal first and second generation antipsychotics. Cases were matched for sample pH, age, post-mortem interval (PMI), and RNA integrity number (RIN) (Table 1). Details of tissue characterization have been previously described [22]. All research was approved by and conducted under the guidelines of the Human Research Ethics Committee at the University of New South Wales (HREC 12435- Investigation of schizophrenia pathogenesis using post-mortem brain tissue). 300 mg of DLPFC was weighed out for total RNA extraction using TRIzol® Reagent (Life Technologies Inc., Grand Island, N.Y., U.S.A., catalogue number: 15596–018), as previously described [23], The quantity and quality of RNA was determined using a spectrophotometer (Nanodrop ND-1000, Thermo Fisher Scientific) and Agilent Bioanalyzer 2100 (Agilent Technologies, Palo Alto, CA, USA).

**Table 1:** Control and Schizophrenia Cohort Demographics.

	Control Group	Schizophrenia Group
Number of Cases	Healthy Controls = 37	SZ = 30, SA = 7
Age (years)	51.1 (18–78)	51.3 (27–75)
Gender	F = 7, M = 30	F = 13, M = 24
Hemisphere	L = 14, R = 23	L = 20, R = 17
pH	6.66 ± 0.29 (5.84–7.19)	6.61 ± 0.30 (5.69–7.09)
Post-Mortem Interval (hours)	24.8 ± 10.97 (6.5–50)	28.8 ± 14.07 (5–72)
RNA Integrity Number (RIN)	7.3 ± 0.57 (6.0–8.4)	7.3 ± 0.58 (6.2–8.4)
Manner of Death	Natural = 37	Natural = 29, Suicide = 8
Age of onset (years)	-	23.7 ± 0.1
Duration of Illness (years)	-	27.6 ± 2.3
Daily Chlorpromazine Mean (mg)	-	692 ± 502
Last Recorded Chlorpromazine Dose (mg)	-	542 ± 374

Key: SZ = schizophrenia, SA = schizoaffective; F = Female, M = Male; L = left, R = Right; ± = Standard Deviation

cDNA derived from total RNA from the DLPFC tissue of a cohort of 20 schizophrenia/schizoaffective cases (referred to as schizophrenia) and 20 control samples was sequenced using the ABI SOLiD platform as previously described [24]. In this study, we took the raw data generated from 19 of the schizophrenia samples and 19 control samples. We excluded one schizophrenia sample as the raw data file had been damaged and it was not possible to use it in further mapping. We also excluded one control sample who was phenotypically male but putatively XXY. We mapped the 50 nucleotide reads to the human genome (hg19) using TopHat2 (v 2.0.4) [25], which calls the Bowtie aligner (v 0.12.8) [26], allowing up to 2 bp mismatches per read (default position). HTSeq-count (Python package HTSeq, python v 2.7.3) was used to generate counts of reads uniquely mapped to known and annotated genes (freeze date October 2011) using the Ensembl annotation file GRCh37.66_chr.gtf (mode = union,–t = exon,–i = gene_name). The count table of uniquely mapped reads was then used for differential expression analysis. Differential expression was tested using the Bioconductor package, edgeR (v 3.12.1) [27] and confirmed using DESeq2 (v 1.10.1) [28]. We used a generalized linear model (GLM) with batch as well as the diagnostic (schizophrenia versus control) as factors in the design matrix [29] in each of the analyses.

In carrying out this analysis, we have used read data (fastq files) obtained from RNA-Seq previously performed on the DLPFC of post-mortem brain. Tools for the analysis of RNA-Seq data have improved rapidly over recent years. Since the time when this data was first analyzed [24] commonly used analysis tools such as edgeR and DESeq have undergone important developments. They now allow covariates such as batch to be routinely incorporated in experimental analysis and provide a more sophisticated treatment of the variation in gene expression (dispersion estimation) [29, 30]. We have used these new methods in the work reported here.

In the edgeR analysis, low count transcripts were excluded and only those genes with at least 1 count per million (cpm), in at least 10 samples, were used for analysis. This filtering retained 17,483 of the original 42,358 transcripts. Normalization was performed using the trimmed mean of M values (TMM) [27]. The dispersion parameter for each gene was estimated with the Cox-Reid common dispersion method [29]. Testing for differential expression employed a negative binomial generalized linear model for each gene.

In the DESeq2 confirmatory analysis, normalization was performed using the median-of-ratios method [28]. Dispersions were estimated using a Cox-Reid adjusted profile likelihood and the Wald test for significance of GLM was used. DESeq2 invokes automatic filtering to optimize the number of genes that have an adjusted p value below the threshold (default 0.1). This resulted in the retention of 17,447 transcripts. In both workflows the Benjamini-Hochberg correction was used to correct for multiple comparisons with a false discovery rate of 0.10.

The differential expression between schizophrenia and control are distinct from those obtained in our earlier analysis [24] due to the alternative analysis streams employed. For this edgeR analysis, estimates of dispersion take into account the actual variation seen in counts for a gene across samples (tagwise dispersion) as well as the common dispersion, which is a value derived from the entire gene set. The tagwise dispersion for a gene is modulated towards the common dispersion by applying a weighting factor (prior.n). Earlier versions of edgeR set the prior.n value at 10, which moved the tagwise dispersions strongly towards the common dispersion value. This was based upon the assumption that RNA-Seq projects generally consisted of few samples and accordingly the small sample size could not alone provide a reliable estimate of dispersion. Later versions of edgeR including v 3.12.1 (used in this analysis) altered these general settings to allow greater sensitivity to the number of samples used in an experiment. The greater the number of samples, the more reliable the tagwise dispersion should be, thus reducing the modulation towards a common dispersion value. The weighting towards the common dispersion is now calculated taking account of the number of samples and groups being analysed. Under the current default settings in edgeR (v 3.12.1) the prior.df is set to 10 with a resulting prior.n of approximately 0.3 (prior.n = prior.df /residual.df). That is, for a data set of this size, with 19 individual samples, there is very little smoothing towards a common dispersion value. Instead, more weight is given to the actual variation in count numbers for a particular gene gleaned from the data for that gene. In a data set where there is a large divergence of count values for a particular gene or where there may be one or two samples with an extreme value, tagwise dispersion will make it less likely that such a gene will be called as differentially expressed. The fact that we see a reduction in differentially expressed genes using the current method is indicative of a spread of gene counts rather than a tighter clustering of values among individuals for a gene expression level.

Clustering functions available in the gplots package in the R environment (v 3.0.2) (http://www.R-project.org) [31] were used to generate heatmaps. The Metacore database was used for ascertaining interaction partners of particular genes. The Cytoscape software platform (www.cytoscape.org) [32] was used for constructing protein interaction networks.

### qPCR analysis

SuperScript® II/III First-Strand Synthesis System (Life Technologies, catalogue number: 11904-018/18080-400) was used for cDNA synthesis from 3 μg RNA. The protocol for SuperScript® II was followed by adding random hexamers, nucleoside triphosphate (dNTP), and RNase OUT to each sample. After incubating at 65°C, tris-hydrochloride (tris-HCl), potassium chloride (KCl), magnesium chloride (Mg2Cl), dithiothreitol (DTT) and SuperScript® II were added. All samples were incubated at room temperature for 10 min before heating it to 42°C for 50 min, followed by 70°C for 15 min. RNase H was added to all samples before incubating at 37°C. We followed the protocol for SuperScript® III First-Strand Synthesis System according to manufacturer’s instructions.

cDNA was plated out with a seven-point serial diluted standard curve, followed by quantitative real time PCR, probing with various primers to amplify members of the nuclear receptor superfamily (Table 2). Samples were measured in triplicates on the 7900HT Fast Real-Time PCR System. The quantity means obtained from the relative standard curve method from serial dilutions of cDNA (1:3, 1:9, 1:27 etc) of our genes of interest were normalized to the geometric mean of four housekeeping genes: β-actin, ubiquitin C, glyceraldehyde-3-phosphate dehydrogenase, and TATA box binding protein (Table 2). There was no difference in the mRNA levels for housekeepers between the schizophrenia and control groups [22].

**Table 2:** List of Taqman genes of interest.

Gene	Gene Name	Assay ID
NR4A1/Nur77	Nuclear receptor subfamily 4, group A, member 1	Hs00374226_m1
NR4A2/Nurr1	Nuclear receptor subfamily 4, group A, member 2	Hs00428691_m1
NR4A3/Nor1	Nuclear receptor subfamily 4, group A, member 3	Hs00545009_g1
KLF4	Kruppel-like factor 4	Hs00358836_m1
VDR	Vitamin D receptor	Hs01045840_m1
RARA	Retinoic Acid Receptor, alpha	Hs00940446_m1
RARB	Retinoic Acid Receptor, beta	GCAGAGCGTGTAATTACCTTGAA/GTGAGATGCTAGGACTGTGCTCT
RARG	Retinoic Acid Receptor, gamma	Hs01559234_m1
RXRA	Retinoid X Receptor, alpha	Hs01067640_m1
RXRB	Retinoid X Receptor, beta	Hs00232774_m1
RXRG	Retinoid X Receptor, gamma	Hs00199455_m1
ACTβ*	Actin, beta	Hs99999903_m1
UBC*	Ubiquitin C	Hs00824723_m1
GAPDH*	Glyceraldehyde-3-phosphate dehydrogenase	Hs99999905_m1
TBP*	TATA box binding protein	Hs00427620_m1

*Housekeeper genes

### Statistical analysis of qPCR results

Normalized data was analyzed using IBM SPSS Statistics 23.0. KLF4 and RXRB were log10 transformed for normal distribution within each diagnostic group. All data were tested for correlation with age, pH, PMI and RIN. The correlations between the gene expressions with each of these factors are listed in S1 Table. ANOVA and ANCOVA were performed when appropriate. The results were analyzed for diagnostic and gender differences. We performed Spearman’s correlation in the schizophrenia group with chlorpromazine dosages, illness duration, and on target mRNAs.

## Results

### Nuclear receptor family genes are expressed in three clusters

**Fig 1:**
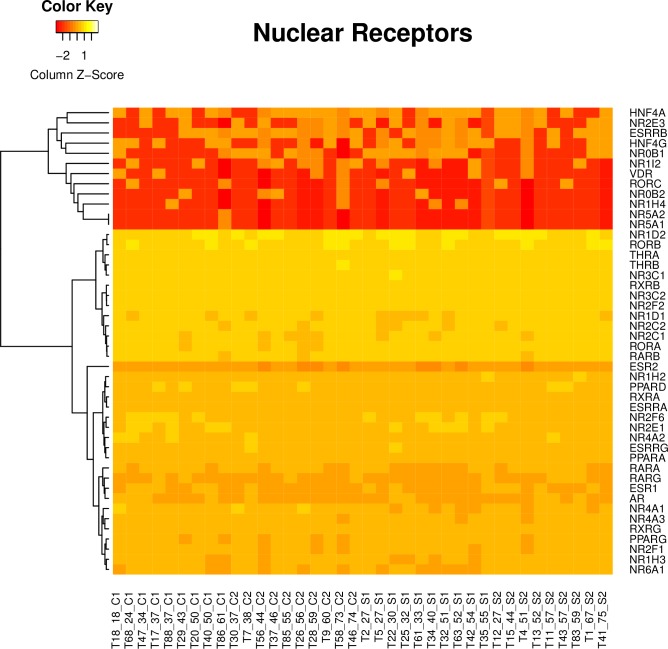
Hierarchical clustering of the NR genes, according to their expression. Heatmap from hierarchal clustering of the NR genes in all samples (19 schizophrenia samples and 19 controls), produced using the heatmap.2 function of the gplots package in R. The samples (controls and schizophrenia) are on the x-axis and the genes are on the y-axis. The CPM values produced by edgeR were adjusted by firstly dividing by the gene length, they were then log2 transformed. The rows (gene names) are clustered and the genes re-ordered (Rowv = T, Colv = F, scale = “column”) resulting in 3 clusters (lowly expressed genes: red, moderately expressed genes: orange, highly expressed genes: yellow).

We sought to characterize nuclear receptor mRNA change in the context of the expression landscape of all nuclear receptor genes in the DLPFC. A cluster-based analysis revealed that the nuclear receptor genes expressed in the adult human prefrontal cortex fall into three main groups, highly expressed genes, moderately expressed genes and lowly expressed genes (Fig 1). Our analysis indicates that NR4A1 falls within the moderately expressed cluster and is expressed at similar abundance levels as the other members of this sub-family NR4A2 (Nurr1) and NR4A3 (Nor-1). Further, the expression levels of the NR4A genes is in a similar range to that of members of the retinoid receptors (RARs and RXRs, part of the NR1B and NR2B sub-families), and the sex steroid hormone receptors such as the estrogen and androgen receptors (ESR1 and AR). As we were interested in seeing the relative expression levels of the nuclear receptors in the DLPFC we adjusted for gene length and also clustered by gene name rather than by sample.

### Nuclear receptor NR4A1 is significantly downregulated in schizophrenia

Using RNA-Seq, we analyzed gene expression in the DLPFC of 19 schizophrenia patients and compared that to 19 controls. The biological coefficient of variation (BCV) calculated using the methods available in edgeR produced a value of 0.3863 for the 19 control samples and 0.479 for the 19 schizophrenia samples. This does indicate a slightly greater degree of variability in samples from people with schizophrenia compared to samples from the controls; however it also reflects that there is also quite a bit of variability in samples from controls as well. Considerable gene expression variability was seen between individuals, which was unsurprising for human brain and patient-derived samples. This could be due to the uncontrolled factors in case-control studies (such as age at death, time of death, or gender) and also due to the heterogeneous nature of schizophrenia. Consequently, we did not see a strong distinction between the two diagnostic categories when examining global gene expression via a multidimensional scaling (MDS) plot (S1 Fig). However, a small group of genes, which had not been previously reported by us to be associated with schizophrenia, was revealed with these novel analysis parameters. The top 20 differentially expressed genes (FDR<0.1) found using edgeR are given in Table 3. S2 Fig in the Supplementary material shows the sensitivity of the number of differentially expressed genes (found at an FDR<0.1) to changes in prior.n from 0.3 to 2, 5 and 10. Full details of the DEGs found using these different values of prior.n are included as S2 Table.

**Table 3:** Significant differentially expressed genes in schizophrenia compared to controls (FDR < 0.1) from use of edgeR.

Gene	Protein name	log2FC^a^	p-value^a^	FDR^a^
NR4A1	Nuclear receptor subfamily 4, group A member 1	-1.13	1 x 10^−6^	0.019
KLF4	Kruppel-like factor 4	-0.99	6.49 x 10^−6^	0.062
EIF2AP4	Eukaryotic translation initiation factor 2A pseudogene 4	1.08	2.26 x 10^−5^	<0.1
RTN4R	Reticulon 4 receptor	-0.69	2.49 x 10^−5^	<0.1
COL5A3	Collagen, type V, alpha 3	-0.55	3.44 x 10^−5^	<0.1
ARRDC3	Arrestin domain containing 3	0.64	3.52 x 10^−5^	<0.1
ADAMTS9-AS2	ADAMTS9 antisense RNA 2	0.53	3.74 x 10^−5^	<0.1
GAREML/FAM59B	GRB2 associated, regulator of MAPK1-like	-0.47	4.52 x 10^−5^	<0.1
MMD2	Monocyte to macrophage differentiation-associated 2	-0.49	5.26 x 10^−5^	<0.1
DUSP1	Dual specificity phosphatase 1	-0.7	5.6 x 10^−5^	<0.1
OAS2	2'-5'-oligoadenylate synthetase 2, 69/71kDa	-0.67	6.98 x 10^−5^	<0.1
ALDH1L2	Aldehyde dehydrogenase 1 family, member L2	0.37	7.82 x 10^−5^	<0.1
ZNF385A	Zinc finger protein 385A	-0.49	8.26 x 10^−5^	<0.1
ZNF610	Zinc finger protein 610	0.4	8.71 x 10^−5^	<0.1
PAPOLB	Poly(A) polymerase beta (testis specific)	0.89	8.98 x 10^−5^	<0.1
PPP1R3B	Protein phosphatase 1, regulatory subunit 3B	0.66	8.99 x 10^−5^	<0.1
SNORD116-24	Small nucleolar RNA, C/D box 116–24	0.64	9.02 x 10^−5^	<0.1
ALDH3A2	Aldehyde dehydrogenase 3 family, member A2	0.3	9.64 x 10^−5^	<0.1
GABRE	Gamma-aminobutyric acid (GABA) A receptor, epsilon	1.2	9.79 x 10^−5^	<0.1
GBAP1	Glucosidase, beta, acid pseudogene 1	-0.57	1 x 10^−4^	<0.1

^a^Calculated in edgeR as described in Methods

In this analysis, the most significant differentially expressed gene was the nuclear receptor NR4A1 (Nur77), for which expression is reduced in schizophrenia (54%, p<0.01, FDR<0.1). The next most significant gene expression change was KLF4 (kruppel-like factor 4), which is reduced by a similar amount in schizophrenia (50% p<0.01, FDR<0.1). We confirmed the edgeR results using a different analysis program, DESeq2, which also found NR4A1 and KLF4 as the most significantly differentially expressed genes with decreases in schizophrenia similar to that found with edgeR (51% and 47%, respectively) (S2 Table).

**Fig 2:**
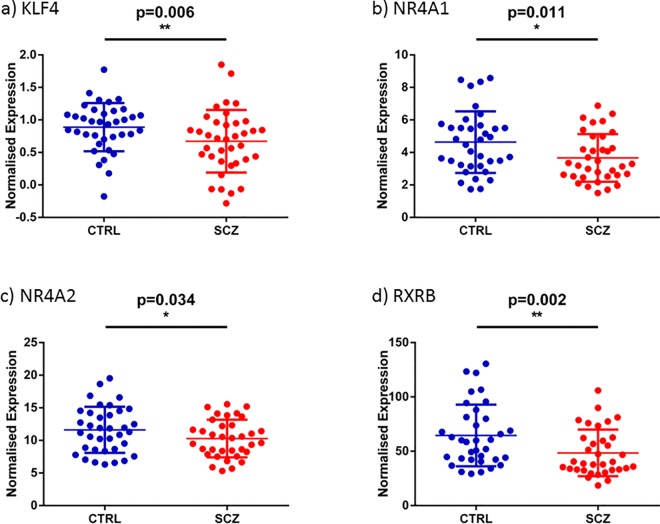
Diagnostic difference of nuclear receptors and KLF4. Graphs show the distribution of gene expression of a) KLF4 b) NR4A1 c) NR4A2 and d) RXRB normalized by the geomean of four housekeeper genes. Blue circles represent individual 37 control samples, and red circles represent the individual 37 schizophrenia samples, all showing mean and standard error of mean (SEM). * represents significance.

To further validate these changes, quantitative PCR was used to measure the expression of these genes in the complete cohort of 74 individuals (37 schizophrenia and 37 controls). This established that both genes were significantly decreased in schizophrenia, (KLF4: F(1,69) = 8.101, p<0.01); NR4A1: F(1,68) = 6.912, p = 0.01; Fig 2, Table 4).

**Table 4:** Results from RT-qPCR analysis.

Gene	F-value	p-value	df	Mean Normalized Expression for Control Group		Mean Normalized Expression for Schizophrenia Group		Percentage Change (%)
NR4A1/ Nur77	F(1, 68) = 6.912	0.011	1	4.634	(n = 36)	3.661	(n = 35)	-20.99
NR4A2/ Nurr1	F(1, 66) = 4.655	0.035	1	11.624	(n = 36)	10.285	(n = 35)	-11.52
NR4A3/ Nor1	F(1, 68) = 1.030	0.314	1	16.204	(n = 35)	14.994	(n = 36)	-7.47
KLF4*	F(1, 69) = 8.101	0.006	1	0.864	(n = 36)	0.609	(n = 35)	-37.36
VDR	F(1, 70) = 0.209	0.649	1	26.381	(n = 37)	25.372	(n = 35)	-3.83
RARA	F(1, 66) = 0.400	0.529	1	2.26	(n = 35)	2.324	(n = 35)	2.83
RARB	F(1, 66) = 0.046	0.5	1	11.038	(n = 33)	11.405	(n = 35)	3.32
RARG	F(1, 62) = 2.824	0.098	1	8.457	(n = 32)	7.254	(n = 34)	-14.23
RXRA	F(1, 66) = 0.744	0.391	1	17.36	(n = 35)	19.049	(n = 34)	9.73
RXRB*	F(1, 66) = 10.256	0.002	1	1.772	(n = 35)	1.647	(n = 34)	-24.99
RXRG	F(1, 66) = 1.669	0.201	1	8.953	(n = 35)	8.035	(n = 35)	-10.25

*Log_10_ transformed data

### qPCR analysis of the other NR4A sub-family and retinoid receptors

**Fig 3:**
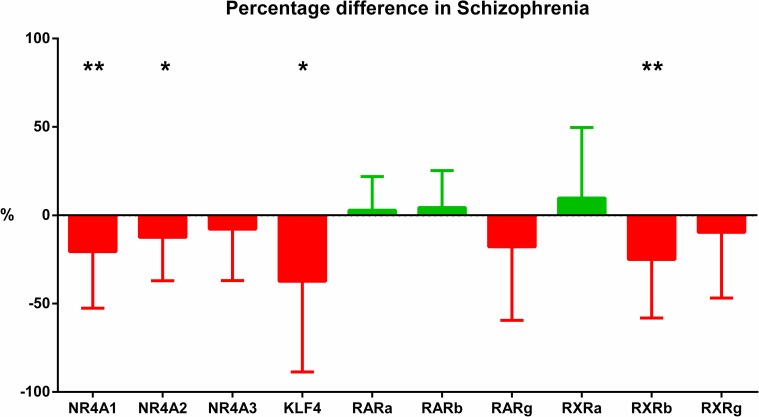
Percentage difference in expression of genes. Overview of the percentage change of NR4A1, NR4A2, NR4A3, KLF4, RARA, RARB, RARG, RXRA, RXRB, and RXRG normalized expressions compared to controls. Red bars indicate the percentage decrease, green bars indicate the percentage increase, all showing standard error of mean (SEM). * represents significance.

*U*sing qPCR in the expanded cohort (37 schizophrenia and 37 controls), we confirmed decreased expression of NR4A1 and also found a significant decrease in NR4A2 expression in schizophrenia (NR4A2: F(1,66) = 4.655, p<0.05) and RXRB: F(1,66) = 10.256, p<0.01; Fig 2, Table 4). We found no significant difference for NR4A3 (F(1,68) = 1.03, p>0.05) or the RARs (A, B, and G), RXRA or RXRG (all F<2.8, p>0.05, Table 4). Percentage differences in gene expression in all 10 targets examined here in the whole sample by qPCR are shown in Fig 3, and a comparison between control and schizophrenia groups of each of the gene expressions are shown in S3 Fig.

### Correlation of gene expression among transcription factors

We performed Pearson’s correlations of the mRNA expression found by qPCR for NR4 sub-family and the retinoid receptors in our extended cohort. Correlations were performed across the combined group of controls and schizophrenia patients. We found that NR4A1 mRNA was significantly correlated with the two other closely related mRNAs NR4A2 and NR4A3. RXRG mRNA was significantly correlated with the other two RXRs (A and B). RXRB mRNA was also correlated with RARG mRNA and NR4A3 mRNA was correlated with RARA mRNA (Table 5).

**Table 5:** Correlation of gene expression for nuclear receptors.

Gene	Gene	N	p-value	FDR Adjusted
NR4A1	NR4A2	68	1.05 x 10^−4^	0.002
NR4A1	NR4A3	69	1.89 x 10^−15^	1.04 x 10^−13^
NR4A3	RARA	67	0.003	0.025
RXRA	RXRG	66	0.003	0.026
RXRB	RARG	63	4.62 x 10^−10^	1.27 x 10^−8^
RXRB	RXRG	66	7.9 x 10^−4^	0.011

### Correlation between gene expression and age

We found a negative correlation between expression of the NR4A family genes and age (NR4A1: r(71) = -0.419, p = 0.0003; NR4A2: r(71) = -0.515, p = 0.000004; NR4A3: r(71) = -0.330, p = 0.005; S4 Fig). This correlation in age was found in both control and schizophrenia groups, with the correlation effect stronger in the control group (S3 Table). There was no significant correlation between age and KLF4 or any of the retinoid receptors mRNAs.

### Correlation between gene expression, and chlorpromazine dosage

**Fig 4:**
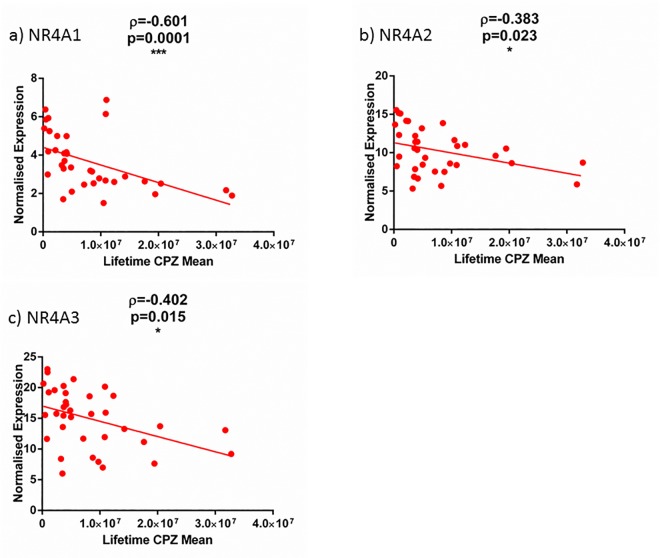
Correlation with Lifetime Chloropromazine treatment. Normalised expression of a) NR4A1 b) NR4A2 and c) NR4A3 correlated against the mean lifetime dosages of chloropromazine.

We found a significant negative correlation between daily chlorpromazine dosage with NR4A1 and NR4A3 (NR4A1: rho(35) = -0.594, p = 0.0002; NR4A3: rho(36) = -0.438, p = 0.008) and no significant correlations between the last recorded chlorpromazine dosage to any of the mRNAs measured. There was a significant negative correlation between estimated lifetime chlorpromazine dosage and expression of NR4A1, NR4A2 and NR4A3 mRNAs (NR4A1: rho(35) = -0.601, p = 0.0001; NR4A2: rho(35) = -0.383, p = 0.023; NR4A3: rho(36) = -0.403, p = 0.015; Fig 4). We did not find a significant correlation with any measure of lifetime chlorpromazine and the retinoid receptor mRNAs measured (rho<0.182, p>0.05).

We also found significant negative correlations between NR4A1 and NR4A2 mRNAs with duration of illness, and a trend between NR4A3 mRNA levels and duration of illness (NR4A1: rho(35) = -0.461, p = 0.005; NR4A2: rho(35) = -0.372, p = 0.028; NR4A3: rho(36) = -0.310, p = 0.067). We did not find a significant correlation between duration of illness and any retinoid receptors mRNAs measured (rho<0.240, p>0.05).

Because there were significant correlations between the NR4A genes with age, we re-analyzed correlations between the NR4A mRNA expressions with illness duration and lifetime dosage in a partial correlation, factoring for age. We found NR4A1 mRNA expression remained significantly correlated with the estimated lifetime antipsychotic dosage (r(32) = -0.401, p = 0.019) and a trend in NR4A3 (r(33) = -0.311, p = 0.069).

### Two-way ANCOVA of diagnosis with gender

We found there was a decrease of RXRG mRNA in females with schizophrenia (F(1,64) = 4.97, p = 0.029) and of RARG mRNA in females with schizophrenia (F(1,60) = 3.942, p = 0.05; S5 Fig). There was no significant change in all other gene targets when we analyzed by two-way ANCOVA of diagnosis and gender.

### Interaction network

**Fig 5:**
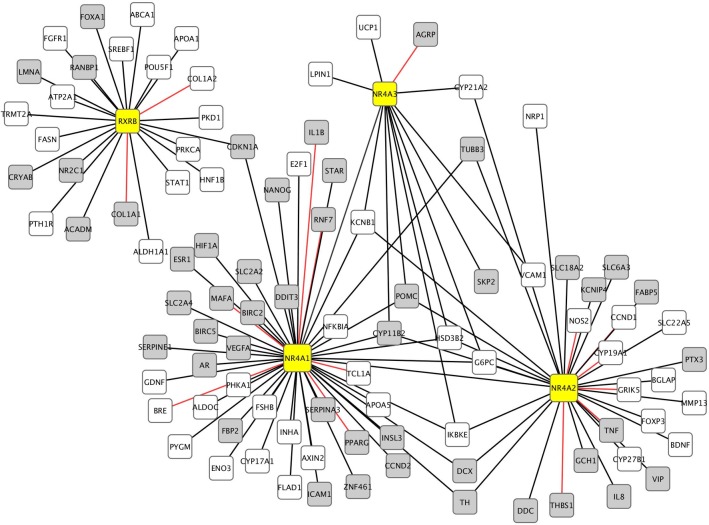
Network map of genes transcriptionally regulated by the NR4A family and RXRB. The Metacore database was used to generate lists of genes transcriptionally activated or inhibited by NR4A1, NR4A2, NR4A3, and RXRB as supported by experimental evidence. The interactions were mapped using Cytoscape. Black lines represent transcriptional activation, red lines represents transcriptional inhibition. The color of the node reflects the trend in expression change in schizophrenia, white (relative decrease in schizophrenia) and grey (relative increase in schizophrenia). It should be noted that these changes may not have reached statistical significance after multiple testing correction.

To investigate the biological effect of NR4A1, NR4A2 and RXRB down-regulation, we generated a network representation of the genes annotated as being transcriptionally regulated by the NR4 sub-family or RXRB (Fig 5). The downstream genes presented in Fig 5 are involved in a broad range of cellular functions, some of which are listed in S4 Table.

Generally, the annotated interactions involve transcriptional activation, although in some cases there is an inhibitory effect of the transcription factor on the target gene. We see some indication of decreased expression in genes activated by NR4A1, for instance PYGM (phosphorylase, glycogen, muscle) (26% reduction, p<0.001, FDR<0.2), consistent with a decrease in NR4A1 activity while genes thought to be inhibited by NR4A1, such as PPARG (peroxisome proliferator-activated receptor gamma) (30% increase, p<0.005 FDR<0.4), have higher levels in schizophrenia. However, the overall picture is complicated with examples of the converse also being demonstrated. This leads us to suggest that complex mechanisms are involved in the transcriptional regulation of these genes and that down-regulation of the NR4A genes and RXRB alone may not be sufficient to induce all the expected changes in downstream gene expression.

## Discussion

This study presents evidence for down-regulation of the nuclear receptors NR4A1, NR4A2, RXRB, and KLF4 mRNAs in the DLPFC in schizophrenia and evidence of reduced RARG and RXRG expression in females with schizophrenia. This study also found a negative correlation between the expression of NR4A genes and estimated levels of antipsychotic exposure. To our knowledge, this is the first study to report a correlation between clinical lifetime chlorpromazine dose and decreased expression of NR4A genes in the DLPFC. Our finding of decreased expression of NR4A1 and NR4A2 mRNA in a large cohort confirms and expands upon a previous finding in post-mortem schizophrenia in the same area of the brain [10], whereas decrease in KLF4 mRNA overall, and RARG and RXRG mRNAs in females with schizophrenia are reported for the first time.

The therapeutic efficacy of various antipsychotic drugs depends upon antagonism of the D2 dopamine receptors [33]. Schizophrenia patients who are being treated with antipsychotic medication are subjected to constant perturbation of dopamine signaling pathways. In this study, we see a highly significant diagnostic decrease in NR4A1 mRNA in the DLPFC and a lesser decrease in NR4A2 mRNA. We also find a significant negative correlation between the estimated lifetime dose of chlorpromazine and the expression of NR4A genes in the DLPFC. This is consistent with the proposition that NR4 family genes play an important role in the frontal cortex and may be regulated via cortical dopaminergic neurotransmission.

It has previously been reported that NR4A1 and NR4A3 expression increases in the murine prefrontal cortex upon a single administration of chlorpromazine [19]. Maheux et al. studied the effect of a number of typical and atypical neuroleptics on the expression of NR4A1 and NR4A3 in different brain regions. Overall, they found that typical antipsychotics, as distinct from atypical antipsychotics, strongly induce the expression of NR4A1 and NR4A3 in striatal areas associated with control of locomotor functions with strength of induction being correlated with the affinity of the neuroleptic drug for the D2 receptor [19]. Our finding of negative correlation between the mRNA of NR4A1, NR4A2 and NR4A3 with lifetime chlorpromazine dose may appear contrary to this result. However, it also suggests that chronic administration of this medication may have a different effect to acute administration. This is in line with other studies which have reported different effects on NR4A gene expression for acute versus ongoing treatment with antipsychotics. For example, the atypical antipsychotic, Clozapine, has previously been found to affect NR4A1 expression, with acute administration resulting in an increase in NR4A1 mRNA and chronic treatment (treatment over 21 days) resulting in a decrease in NR4A1 expression [18]. Haloperidol also increased NR4A1 expression in the dorsolateral striatum on acute treatment only [18]. To date, there is little evidence that antipsychotic drugs affect RXR gene expression. We found no correlation between RXR expression and chlorpromazine medication. Langlois et al. report that haloperidol had modest effects on RXRG expression in the dorsolateral portion of the striatum without any effect in other regions [34]. Although we found no correlation between retinoid gene expression and chlorpromazine it is possible that medication has an effect on other genes or proteins which interact with or affect the retinoid receptors and contribute to the gene expression changes reported here.

It has been proposed that NR4A1 and RXR work together as adaptive homeostatic regulators of dopamine function by reducing the effect of alterations in dopamine neurotransmission [17]. Given the link between NR4A genes and response to antipsychotic medication it is difficult to say whether NR4A genes are dysregulated in schizophrenia prior to commencement of antipsychotic medication. However, if dopamine signaling dysfunction is involved in the etiology of schizophrenia it is plausible that NR4A genes are vulnerable to changed expression in the development of the condition. Up or down regulation of NR4A genes is likely to have some effect on genes transcriptionally regulated by these factors. A change in NR4A can result in switching the transcriptional pathway between retinoic acid initiated (RARs) and 9-cis retinoic acid (RXRs) initiated programs [35] with potential perturbations in the dopaminergic pathways and in gene expression affected by dopamine.

Functions ascribed to NR4A1 in neuronal differentiation and neurite outgrowth [36, 37], learning and memory and immunity are all potentially relevant to schizophrenia. Notably, neurotrophic factors that promote neuronal survival and growth mediate their effect through receptors such as NR4A1 [38]. NR4A1 was first recognized through its response to nerve growth factor (NGF), which induces neuronal differentiation and neurite outgrowth [37]. Another neurotrophin which has also been found to be reduced in schizophrenia is brain-derived neurotrophic factor [39]. A potential effect on neural plasticity is also consistent with recent work indicating that the NR4A sub-family is involved in the processes of learning and memory [40, 41].

In our analysis, we found all three NR4A receptor mRNA levels to be decreased with age. The role of NR4A receptors in brain aging is currently unknown. With human brain aging, increased DNA damage is found and since NR4A receptors may protect against DNA damage [42, 43] our finding of decreased NR4A receptors with age may contribute to loss of DNA repair in brain cells, as has been observed in damaged skin cells [44]. Another prominent event that occurs as humans age is a decrease in metabolic rate particularly in brain [45, 46] and the down regulation of NR4A synthesis may also play a role in down-regulating cellular metabolism. In support of this, an increase in metabolism is observed in muscle cells overexpressing NR4A receptors [47–49]. Thus, our findings support the hypothesis that increasing NR4A transcription or function could be a potential angle to counteract some of the effects associated with human brain aging as previously proposed [42].

The decrease in RXRB in schizophrenia is interesting as the genetic locus of RXRB (6p21.3) has been linked to schizophrenia [9]. Ablation of RXRB leads to lethality in 50% of embryos indicating an important role for this gene in early development. However, surviving embryos only display mild defects, primarily male infertility related to lipid metabolism defects in Sertoli cells [50]. Mutant mice, with ablation of RXRB-RXRG, RARB-RXRB or RARB-RXRG have shown locomotor defects and decrease of dopamine receptors DR1 and DR2 in the ventral striatum but not in the dorsal striatum [51]. There is evidence that suggests DRD2 is dysregulated in schizophrenia brains [52–54]. Krezel et al. suggest that RXRB and RXRG may be functionally redundant in locomotion control [51], which is considered a functional readout of dopamine activity in the brain.

Our finding of decreased RARG and RXRG mRNA levels in females with schizophrenia may be related to changes found in estrogen and/or estrogen receptors (ER) signaling. Indeed, direct protein interaction between the retinoid receptors and the ERs via their ligand binding domain has been documented [55, 56]. Further, retinoid receptors have been shown to be regulated by estrogen/ER [57] through an estrogen response element (ERE) on the RAR gene promoter [58–61]. RARs and ER can bind to the overlapping DNA sites [62], which may cause antagonism. However, rather than simple antagonism, there can also be cooperation between RARs and ER in the control of gene expression [63, 64]. More work needs to be done to determine the role the retinoid receptors and ER proteins in brain neuropathology and how they may be individually or reciprocally altered in schizophrenia particularly in females.

We have also found KLF4 to be differentially expressed in schizophrenia compared to controls. KLF4 is a transcription factor in the kruppel-like factor family which regulates multiple biological functions and is involved in neurogenesis, neuronal differentiation and neurite outgrowth [65]. KLF4 is regulated by RARA [66], and it can also inhibit RARA [67] in vascular smooth muscle cells in a feedback-loop fashion. KLF4 mRNA and protein expression is found to be increased in skin and breast cancer [68–71]. In skin, RARG and RXRA are found to be antagonists of KLF4 [72]. Interestingly, KLF4 inhibits cell proliferation in the brain [65] and is down-regulated in neurogenesis [73]. It has been found there may be a decreased rate of cell proliferation and decreased neurogenesis in the hippocampus in schizophrenia [74–76]. However, the role of KLF4 in differentiated cells in the cerebral cortex is not well understood.

Transcriptional regulation by the nuclear receptors is complicated by heterodimerization and their activation by multiple ligands. Furthermore, recent genome-wide studies reveal that NR binding regions are enriched for sequence motifs of other transcription factors, such as Sp1, AP-1, and C/EBP motifs, suggesting that NRs interact with other transcription factors to regulate target gene expression [77, 78]. The NRs thus operate in a complex environment that may be tuned to provide specificity in particular tissues, cell types or environments. Quantifying and comparing the mRNA of nuclear receptors contributes to our understanding of their activity. However, a thorough investigation will also require study at the protein level. We and others have previously noted differences between protein and RNA abundance in the nuclear receptors highlighting the role of post-transcriptional regulation in these genes [6, 79]. The task of teasing out the functions of the nuclear receptors in normal and schizophrenia brains and their use as biomarkers in blook could be an area of future research. Another important research question is what cell types are expressing these nuclear receptors.

## Conclusion

This study reports significant changes in the nuclear receptors NR4A1, NR4A2, and RXRB and KLF4 in schizophrenia and provides further evidence of a role for the nuclear receptors in the disease process. Evidence is growing in support of an important role for NR4A1 and NR4A2 in neurogenesis, learning and memory, which may be associated with the role of NR4A family genes in dopaminergic pathways. Cognitive defects and changes to dopamine signaling are well known effects of schizophrenia and of current treatment protocols. These genes also play a role in immune function which is emerging as an important focus in schizophrenia research [24, 80, 81]. More generally this work highlights the role of a subset of nuclear receptors that link environmental cues to the genetic landscape in this complex disease.

## Supporting Information

S1 Fig MDS plot and Venn diagram of DEGs found using edgeR and DESeq2. Multidimensional scaling (MDS) plot of all samples, SCZ samples in batch 1 (orange), Control samples in batch 1 (cyan), SCZ samples in batch 2 (red), Control samples in batch 2 (blue). (B) DEGs were identified using a glm model in both the edger and DESeq2 tools taking account of batch and SCZ, using the default settings for edgeR and DESeq2.
(TIF)

S2 Fig Venn diagram of DEGs found using edgeR with different values of prior.df. The differentially expressed genes calculated using edgeR but varying the parameter prior.df., using the current default setting (prior.df = 10) and increasing this to prior.df = 70 (equivalent to prior.n = 2), and prior.df = 175 (equivalent to prior.n = 5) and prior.df = 350 (equivalent to prior.n = 10).
(TIF)

S3 Fig Normalized expressions of genes by diagnosis. Overview of the normalized expressions of NR4A1, NR4A2, NR4A3, KLF4, RARA, RARB, RARG, RXRA, RXRB, and RXRG. Blue bars indicate control group and red bars indicate schizophrenia group, all showing standard error of mean (SEM). * represents significance.
(TIF)

S4 Fig Correlations with age. Normalized expressions of a) NR4A1 b) NR4A2 and c) NR4A3 correlated against age.
(TIF)

S5 Fig Diagnostic and Gender Differences. Two-way ANCOVA analysis of the normalized expression of diagnosis and gender of a) RARG and b) RXRG.
(TIF)

S1 Table Correlations between gene expressions and correlation factors. (XLSX)

S2 Table a. EdgeR DE analysis using only common dispersion. b. EdgeR DE analysis using df = 350 equating to prion.n = 10. c. EdgeR DE analysis using df = 175 equating to prion.n = 5. d. EdgeR DE analysis using df = 70 equating to prion.n = 2. e. EdgeR DE analysis using current defaults (df = 10).
(ZIP)

S3 Table Correlation between NR4A gene expressions and Age. (XLSX)

S4 Table Functions associated with genes downstream of NR4A family and RXRB. (XLSX)
