# Supplementary figures and images for: Transcriptomic Analysis Shows Decreased Cortical Expression of NR4A1, NR4A2 and RXRB in Schizophrenia and Provides Evidence for Nuclear Receptor Dysregulation
Source: PLoS One. 2016 Dec 16;11(12):e0166944. doi: 10.1371/journal.pone.0166944 (PMC5161508; doi:10.1371/journal.pone.0166944)

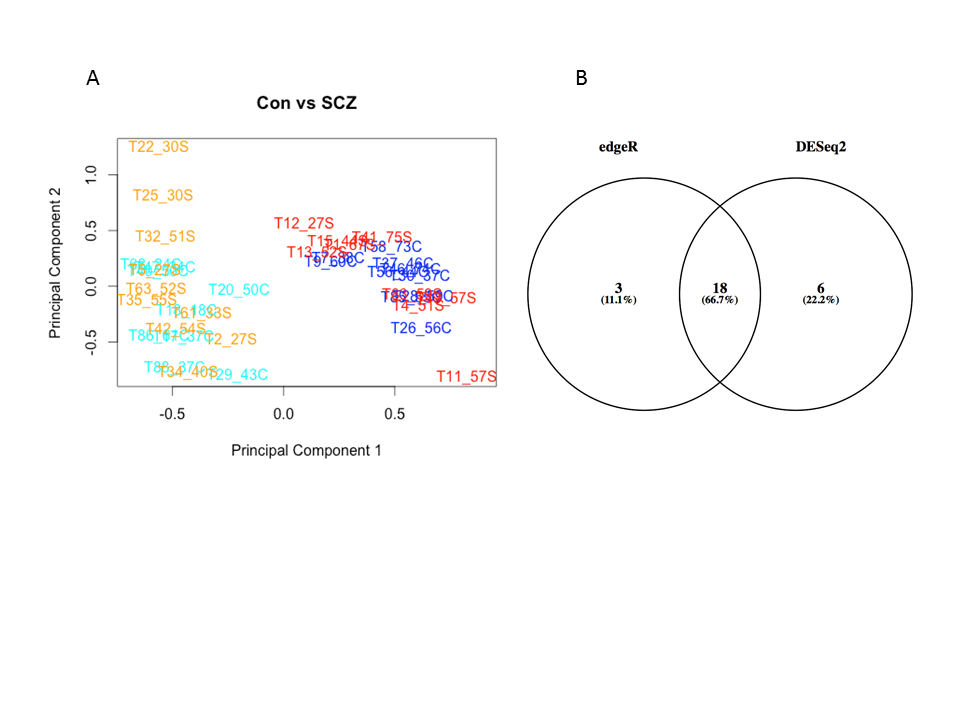

Supplement: S1 Fig — Multidimensional scaling (MDS) plot of all samples, SCZ samples in batch 1 (orange), Control samples in batch 1 (cyan), SCZ samples in batch 2 (red), Control samples in batch 2 (blue). (B) DEGs were identified using a glm model in both the edger and DESeq2 tools taking account of batch and SCZ, using the default settings for edgeR and DESeq2. (TIF) [file pone.0166944.s001.tif]

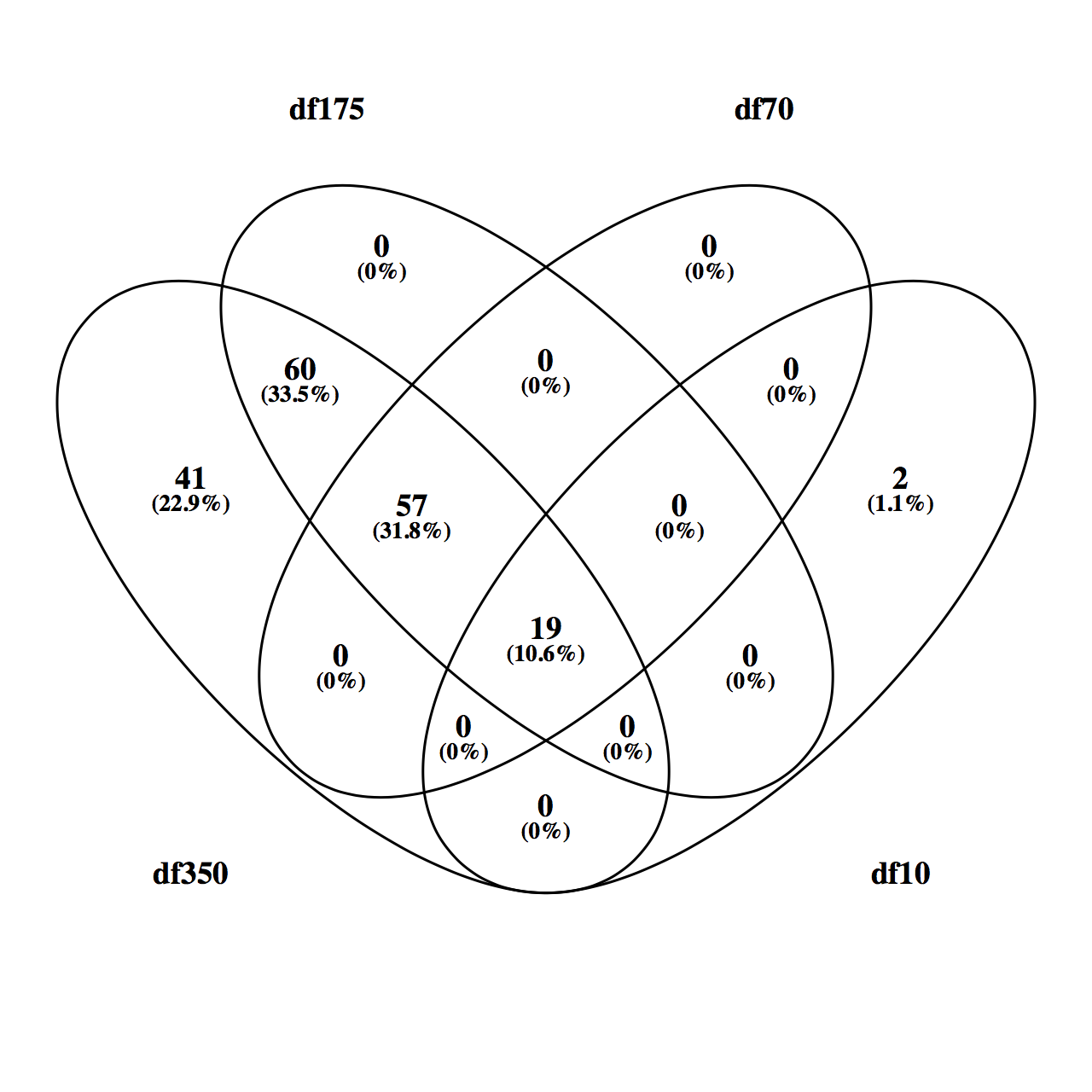

Supplement: S2 Fig — The differentially expressed genes calculated using edgeR but varying the parameter prior.df., using the current default setting (prior.df = 10) and increasing this to prior.df = 70 (equivalent to prior.n = 2), and prior.df = 175 (equivalent to prior.n = 5) and prior.df = 350 (equivalent to prior.n = 10). (TIF) [file pone.0166944.s002.tif]

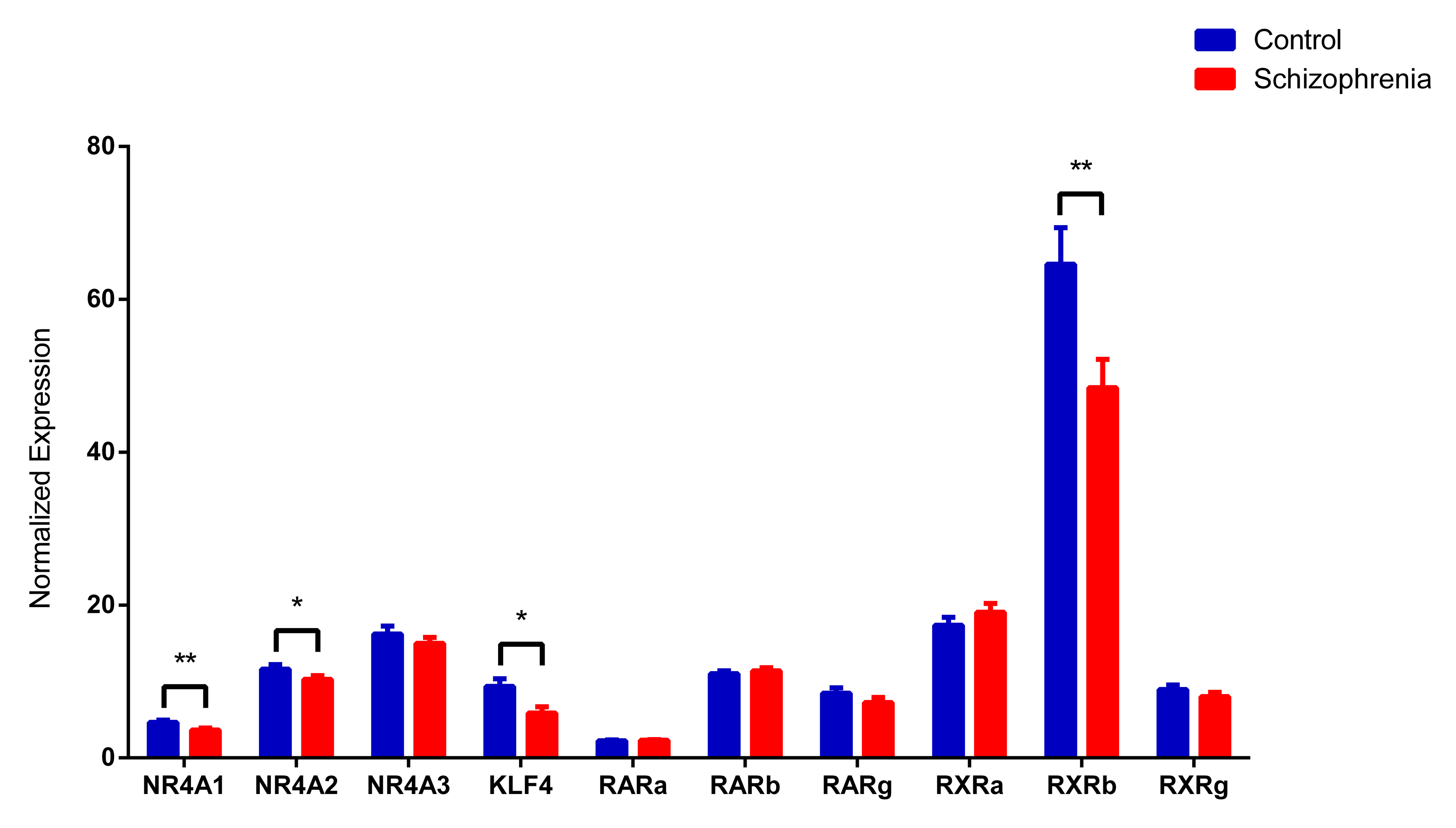

Supplement: S3 Fig — Overview of the normalized expressions of NR4A1, NR4A2, NR4A3, KLF4, RARA, RARB, RARG, RXRA, RXRB, and RXRG. Blue bars indicate control group and red bars indicate schizophrenia group, all showing standard error of mean (SEM). * represents significance. (TIF) [file pone.0166944.s003.tif]

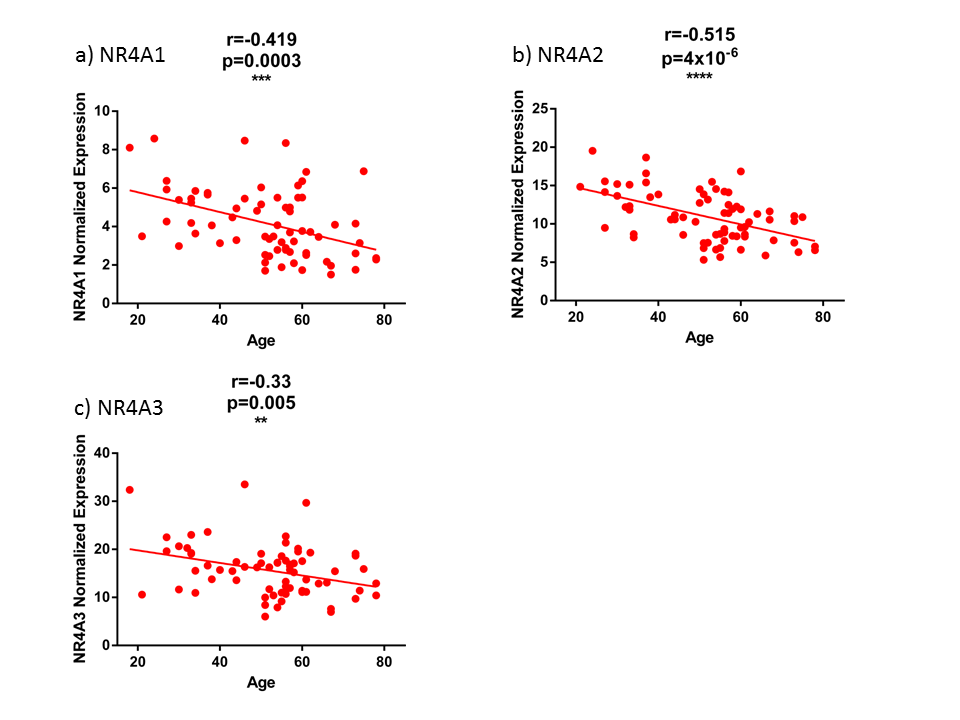

Supplement: S4 Fig — Normalized expressions of a) NR4A1 b) NR4A2 and c) NR4A3 correlated against age. (TIF) [file pone.0166944.s004.tif]

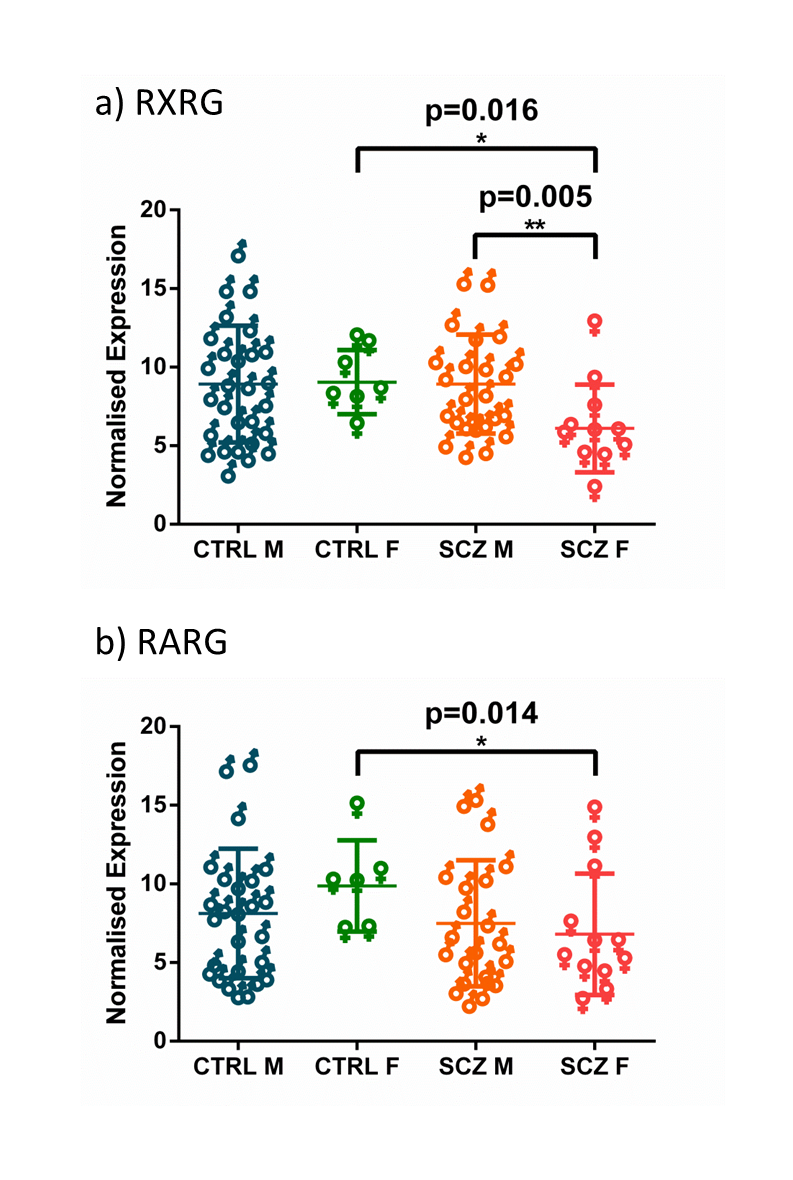

Supplement: S5 Fig — Two-way ANCOVA analysis of the normalized expression of diagnosis and gender of a) RARG and b) RXRG. (TIF) [file pone.0166944.s005.tif]
